# Inhibition of androgen receptor can decrease fat metabolism by decreasing carnitine palmitoyltransferase I levels in skeletal muscles of trained mice

**DOI:** 10.1186/s12986-019-0406-z

**Published:** 2019-11-27

**Authors:** Jisu Kim, Jonghoon Park, Nahyun Kim, Hun-young Park, Kiwon Lim

**Affiliations:** 1 Department of Sports Medicine and Science, Konkuk University, Gwangjin-gu, Seoul Korea; 2 Physical Activity and Performance Institute (PAPI), Konkuk University, Gwangjin-gu, Seoul Korea; 3 Department of Physical Education, Korea University, Seoul, Korea; 4 Department of Physical Education, Konkuk University, Gwangjin-gu, Seoul Korea

**Keywords:** FAT/CD36, CPTI, Androgen hormone, Fat metabolism, LCFA transport protein, Androgen receptor

## Abstract

**Background:**

Androgen hormone levels are strongly associated with obesity in adult mammals, especially with advanced age. We investigated androgen receptor inhibition on fat metabolism and long-chain fatty acid (LCFA) transport proteins in skeletal muscle during exercise.

**Methods:**

Male ICR mice were randomly divided into three groups: CON (control), EX (exercise), and EXIN (exercise + androgen receptor inhibition). EX and EXIN groups were trained on a treadmill five times a week. After 4 weeks, the fat metabolism of each group was measured using open-circuit calorimetry during 1 hour of exercise. After the metabolism measurement, the expression levels of LCFA transport proteins (FAT/CD36, CPTI) were analyzed in skeletal muscle.

**Results:**

Weight gain and final body weight were significantly lower in the EX group than in either the CON or EXIN groups. Conversely, food intake was significantly higher in the EX group than it was in the CON and EXIN groups. The total weight (CON; 2.07 ± 0.6, EX; 1.64 ± 0.2, EXIN; 1.95 ± 0.2) of the abdominal adipose tissue were significantly lower in the EX group than in the CON and EXIN groups (*P* < 0.05). However, there was no different between the CON and EXIN group. Oxygen uptake and fat oxidation during exercise tended to be lower (12%) in the EXIN group than in the EX group. Total fat oxidation in the EXIN group was significantly lower during the initial 20-min (*P* < 0.003) and 40-min (*P* < 0.041) phases compared to that in the EX group. In addition, the level of FAT/CD36 protein in the EX and EXIN groups was approximately double that in the CON group (*P* < 0.001, *P* < 0.001). CPTI expression in the EX group was higher than that in the EX group (*P* < 0.0069) as well as in the CON group.

**Conclusion:**

Exercise training increases the expression of LCFA transport proteins (FAT/CD36, CPTI). Blocking androgen receptors can decreases the expression of CPTI in the skeletal muscle, which reduces fat metabolism. Thus, reducing sex hormones or suppressing the sensitivity of AR receptors can inhibit energy efficiency and fat metabolism by suppressing CPTI.

## Background

Androgen hormones are negatively associated with the central obesity index in older adults [1].. Androgens are important factors that determine body composition in males [2]. Steady increases in body fat mass accompany the age-dependent decrease in serum testosterone levels in men [3]. These morphological features are linked to metabolic dysfunction, and testosterone deficiency is associated with energy imbalance, impaired glucose control, reduced insulin sensitivity and dyslipidemia [4]. Therefore, maintaining higher levels of androgens is important to prevent obesity.

An androgen is any natural or synthetic steroid hormone in vertebrates that binds androgen receptors (AR) to regulate the development and maintenance of male characteristics [5]. ARs, members of the steroid hormone receptor family, play important roles in the physiology and pathology of many tissues [6]. AR ligands, which include circulating testosterone and locally synthesized dihydrotestosterone, bind to and activate ARs to elicit their effects [7, 8]. The AR initiates a diverse range of biological actions that play roles in the development and maintenance of the reproductive, musculoskeletal, cardiovascular, immune, neural, and haemopoietic systems. Aberrant AR signaling may be involved in the development of tumors in the prostate, bladder, liver, kidney, and lung [7, 9].

ARs are present in muscles and brown adipose tissues (BAT) that consume and expend energy [10]. ARs are also expressed in cultured brown adipocytes. We previously reported that blocking androgen hormone production decreases fat oxidation during acute exercise [11]. This study observed metabolism during acute exercise but did not examine the effect of AR inhibition on a long-term exercise training program that would more accurately reflect a general health regimen. We also focused on whole-body metabolism but did not investigate tissue-specific effects.

Guerrero J et al. subjected 9-week-old male CB17SCID mice to an AR inhibitor (enzalutamide; 1–50 mg/kg/day) and measured tumor volume and body weight at 2-to 3-day intervals for 4 weeks [12]. The AR inhibitor (10 and 50 mg/kg/day) treatment decreased tumor volume and increased body weight by 8.5 and 12.1% compared to baseline respectively, which indicated healthy mice. In contrast, 13-to-14 week-old C57BL/6 male mice that underwent chronic (21 days) androgen hormone treatment may have developed an improved metabolic profile by regulating lipolysis and various critical pathways. We therefore hypothesized that androgen hormone enhances fat oxidation and energy expenditure.

Endurance exercise increases capillary density, mitochondrial population, and the activity of the tricarboxylic acid cycle and other oxidative enzymes (hormone-sensitive lipase, catecholamines, β-oxidation enzymes, etc.) [13]. In addition, exercise training demands a supply of energy in the form of long-chain fatty acids (LCFAs) that are provided by transport proteins. It was recently reported that fatty acid translocase/cluster of differentiation 36 (FAT/CD36) and carnitine palmitoyltransferase I (CPTІ) play key roles in muscle fuel selection, exercise performance, and the induced adaptation of fatty acid oxidation in skeletal muscles of humans and animals [14, 15].

The direct effect of AR blockade is understood to be a decrease in resting metabolic rate and a concomitant increase in body weight [12]. We previously found that AR blockade decreased whole body fat utilization during acute exercise. However, this scenario is atypical of clinical reality. A more relevant scenario would be the effect of a chronic AR blockade on energy substrate utilization, comparing a regular exercise regimen to sedentary behavior. We hypothesize that chronic AR blockade in male mice would inhibit the elevation of LCFA transport protein (FAT/CD36 and CPTІ) expression that is normally induced by running training. The physiological effect would be a reduction of whole-body fat oxidation. Accordingly, the purpose of this study was to ascertain the effects of chronic AR blockade on the expression of LCFA transport proteins in skeletal muscle, and on whole-body fat oxidation during exercise.

## Materials and methods

### Animals

Twenty-four male ICR mice were obtained from Orient Bio Inc. (Seongnam, Korea) and adapted to the laboratory housing conditions for 1 week. They were given free access to water and a non-purified commercial diet (5 L79, Orient Bio Inc.) containing crude protein (180 g/kg); crude fat (52 g/kg); crude fiber (52 g/kg); minerals (57 g/kg); and carbohydrates (368 g/kg). The protein, fat, and carbohydrate ratio (%) based on calories was 21:14:65. The gross and metabolizable caloric contents of the diet were 4.04 and 3.21 kcal/g, respectively.

**Fig. 1:**
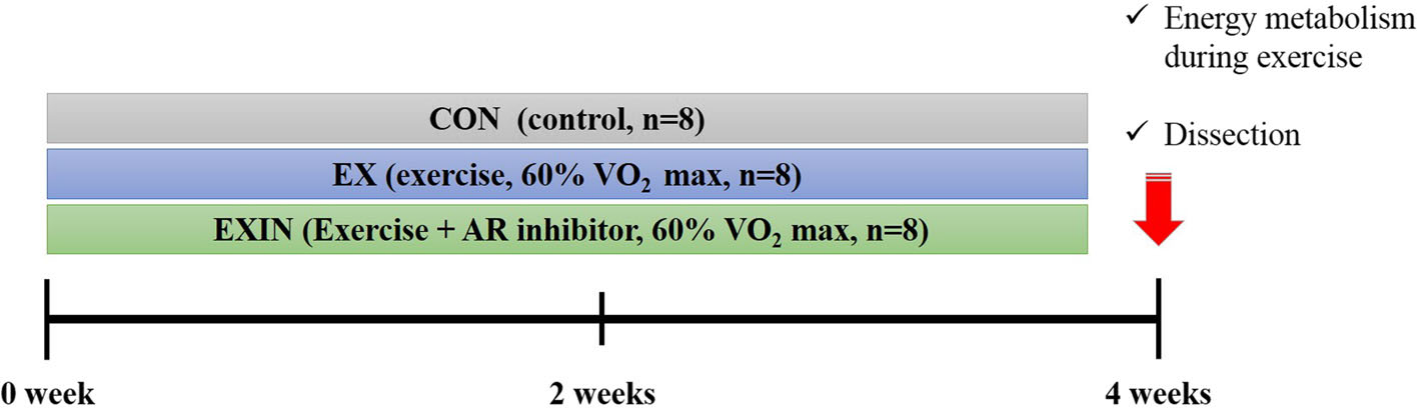
Experimental design. CON (control, *n* = 8), EX (exercise, 60% VO 2 max, n = 8), EXIN (Exercise + androgen receptor (AR) inhibitor, 60% VO 2 max, *n* = 8). EX and EXIN groups underwent training by running on a treadmill five times a week for 4 weeks. After 4 weeks of training, energy metabolism was measured during 1 h exercise using the most recent training conditions (18 m/min, 8° slope, 60% of maximum oxygen uptake). After measurement the mice were euthanized and dissected, and skeletal muscle tissue samples were collected for protein expression analysis using western blotting

At the age of 7 weeks, the mice were randomly divided into three groups: CON (control, *n* = 8), EX (exercise, n = 8), EXIN (exercise + androgen receptor inhibitor; 10 mg/kg, n = 8). Body weight and food intake were measured daily for 4 weeks. EX and EXIN groups underwent training by running on a treadmill five times per week for 4 weeks. The AR inhibitor enzalutamide (Medivation, Inc. San Francisco, CA) was dissolved in mixed solution (2% dimethyl sulfoxide in distilled water). Based on previous studies, AR inhibitor was administered orally every day for 4 weeks [12]. The CON and EX groups received the vehicle (2% dimethyl sulfoxide with distilled water without AR inhibitor) only. Details of the experimental design are shown in Fig. 1.

### Training method

The EX and EXIN groups were adapted to treadmill training intensity of 12 m/min, 8° slope for 3 days. The mice were then trained 5 times per week for 4 weeks with the following conditions: 15 m/min, 8° slope, 50 min/day for 2 weeks; then 18 m/min, 8° slope, 50 min/day (about 60% of maximum oxygen uptake) for 3–4 weeks [16, 17].

### Energy metabolism alterations during exercise

After 4 weeks of training, energy metabolism was measured during 1 hour of exercise using the training conditions of the final week (18 m/min, 8° slope, 60% of maximum oxygen uptake). Two hours before the measurement, the mice were placed in exercise metabolic chambers with a volume of approximately 3 L to reduce stress. The flow rate was kept constant at 3 L/min and measured for 1 h. The energy metabolism during exercise was measured using an open-circuit device based on methods reported in previous studies [17].

### Surgical procedure

After metabolic measurement subjects were euthanized by sodium pentobarbital overdose. Skin was removed from the hind limbs and soleus muscle was extracted by established methods [18].

### Protein extraction and western blot analysis

The muscle (soleus) tissue samples (35 mg) were homogenized in 700 μL EzRIPA lysis buffer (ATTO Biotechnology, Sungnam, Korea) using a mortar and TissueRuptor (QIAGEN, Germany). The muscle lysates were mixed using a rotator for 2 h at 4 °C and then centrifuged at 12,000 rpm at 4 °C for 15 min. The protein concentration of the supernatant was determined using a GenDEPOT protein assay plus reagent kit (Gen-Depot Laboratories, USA) using bovine serum albumin (BSA) as the standard.

Total protein (25 μg/lane) was separated using 12% sodium dodecyl sulfate (SDS)-polyacrylamide gel electrophoresis (PAGE) at 80–110 V for 150 min and then transferred to a polyvinylidene difluoride (PVDF) membrane (Millipore, Billerica, MA, USA) at 100 V for 2 h. The membrane was blocked for 1 h at 25 °C with phosphate-buffered saline (HyClone Laboratories, USA) containing 5% skim milk (Difco, USA) and then washed three times (5, 5, and 15 min) with PBS plus 0.1% Tween 20 (PBS-T) buffer. After an overnight incubation at 4 °C with primary antibodies against FAT/CD36 and CPTІ (Santa Cruz Biotechnology, USA), the membranes were washed with PBS-T and incubated with an HRP-conjugated secondary antibody for 1 h at 25 °C .

Immunodetection was carried out using an enhanced chemiluminescence (ECL) detection reagent (Amersham Biosciences, Uppsala, Sweden). Quantitative analysis was carried out using the Image J program (National Institutes of Health, NIH, Bethesda, MD, USA) including data from at least three independent experiments.

### Blood analysis

Blood samples were collected after for 4 weeks. Plasma glucose was measured using commercial kits (Asan Pharmaceutical Co.,Hwaseong-si Gyeonggi-do, Korea), the plasma FFA level using a non-esterified fatty acid kit (Wako Pure Chemical Industries), the plasma insulin level was determined with an enzyme-linked immunosorbent assay kit (Morinaga Bioscience Laboratory, Yokohama, Japan), and the plasma glycerol level was determined using the colorimetric assay kit (Cayman CO., Ellsworth RD, USA) according to the instruction of the manufacturer.

### Statistical analysis

Data are given as means ± standard deviation (SD). All statistical analyses were performed with SPSS version 19.0 software (SPSS, Inc., Chicago, IL, USA). Oxygen uptake, RER (respiratory exchange ratio), carbohydrate oxidation, fat oxidation, food intake, and body weight were analyzed by two-way repeated measures analysis of variance (ANOVA). One-way ANOVA was used to determine the changes in the sums of the energy metabolism during exercise, body weight, and food intake. Least squares difference (LSD) post-hoc analysis was conducted if significance was obtained. Differences were considered significant at *P* < 0.05.

## Results

### Changes in body weight, food intake and abdominal fat

Table 1 shows the changes in body weight, food intake and abdominal fat in CON, EX and EXIN groups after 4 weeks of treatment and endurance training. There were significant differences between the groups in final body weight (CON; 40.51 ± 1.8, EX; 36.14 ± 1.1, EXIN; 40.01 ± 1.3) and weight gain (6.97 ± 2.0, 3.30 ± 1.50, 6.98 ± 2.0). The EX group values were significantly lower than the CON and EXIN groups (*P* < 0.001, *P* < 0.001). However, the EXIN group underwent the same exercise intensity as the EX group but did not lose weight, gaining a similar amount as the CON group (*P* = 0.619). Nevertheless, food intake (in g/4 weeks and g/day) was significantly higher in the EX group than in the CON and EXIN groups (*P* < 0.001, *P* < 0.002). The total weight (CON; 2.07 ± 0.6, EX; 1.64 ± 0.2, EXIN; 1.95 ± 0.2) of the abdominal adipose tissue were significantly lower in the EX group than in the CON and EXIN groups (*P* < 0.05). However, there was no different between the CON and EXIN groups. In additional, the mesentery fat was significantly higher in the EXIN group than in EX group (*P* < 0.05). However, there was no significant different between the EXIN and CON groups. Furthermore, The EX group tended to have less abdominal fat than other groups. On the other hand, the EXIN group showed similar fat weight as the CON group without exercise.

**Table 1:** Change of body weight, food intake and abdominal fat for 4 weeks of experiment

		CON	EX	EXIN
BW (g)	Initial	33.53 ± 1.8	32.81 ± 1.4	32.96 ± 0.9
	Final	40.51 ± 2.0^a^	36.14 ± 1.1^b^	40.01 ± 1.3^a^
	Gain	6.97 ± 2.0^a^	3.30 ± 1.5^b^	6.98 ± 2.0^a^
Food intake (g)	Day	5.15 ± 0.1	5.87 ± 0.1	5.60 ± 0.2
	Total	144.20 ± 2.9	164.40 ± 1.5	156.83 ± 4.1
Abdominal fat (g)	Epididymal	0.93 ± 0.3^a^	0.69 ± 0.1^b^	0.81 ± 0.2^ab^
	Perirenal	0.36 ± 0.1	0.25 ± 0.0	0.33 ± 0.1
	Mesentery	0.78 ± 0.1^ab^	0.69 ± 0.0^b^	0.81 ± 0.1^a^
	Total	2.07 ± 0.6 ^a^	1.64 ± 0.2 ^b^	1.95 ± 0.4 ^ab^

*Note.* Change of body weight (g), food intake (g/day, g/4 weeks) and abdominal fat for 4 weeks. CON (control, *n* = 8), EX (exercise, 60% VO_2_ max, *n* = 8), EXIN (Exercise + AR inhibitor, 60% VO_2_ max, *n* = 8). Values are presented as means ± standard deviations (*n* = 8). Different superscripts mean significant differences among the groups

### Changes in plasma glucose, FFA, insulin and glycerol level

Table 2 shows the changes in plasma glucose, FFA, insulin and glycerol levels. Plasma glucose levels did not change among the groups. However, plasma FFA levels were 25% (*P* < 0.001) and 7% (*P* < 0.05) lower in EX and EXIN groups than in CON groups. In additional, plasma glycerol levels were 2.17-fold (*P* < 0.001) and 1.7-fold (*P* < 0.001) higher in EX group, respectively compared to respective CON and EXIN groups. However, no significant different between the CON and EXIN groups. Insulin levels were lower by 46 and 30% in EX and EXIN groups compared to CON group (*P* < 0.001, *P* < 0.01).

**Table 2:** Change in the plasma glucose, FFA, insulin and glycerol levels

	CON	EX	EXIN
Glucose (mg/dl)	129.3 ± 23.4	123.7 ± 24.3	129.11 ± 20.0
FFA (mEq/L)	0.15 ± 0.2^a^	0.12 ± 0.1^b^	0.14 ± 0.1^b^
Insulin (ng/ml)	1.80 ± 0.5^a^	1.23 ± 0.2^b^	1.38 ± 0.1^b^
Glycerol (mg/L)	8.21 ± 2.6^a^	17.82 ± 5.5^b^	10.44 ± 3.9^a^

Note. Change in the plasma glucose, FFA, insulin and glycerol levels after 4 weeks of exercise. CON (control, *n* = 8), EX (exercise, 60% VO_2_ max, n = 8), EXIN (Exercise + AR inhibitor, 60% VO_2_ max, *n* = 8). Values are presented as means ± standard deviations (*n* = 8). Different superscripts mean significant differences among the groups

### Energy metabolism during exercise

**Fig. 2:**
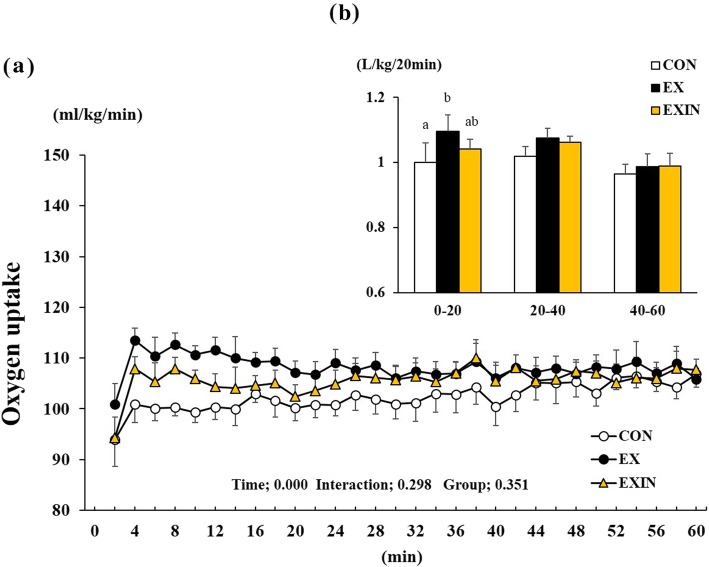
Change in oxygen uptake during 1 h of exercise (**a**), the sum of the oxygen uptake of the test group for each 20 min block of exercise (**b**). Energy metabolism measured during 1 h of exercise. CON (control, *n* = 8), EX (exercise, 60% VO 2 max, *n* = 8), EXIN (Exercise + AR inhibitor, 60% VO 2 max, *n* = 8). Values are presented as means ± standard deviations (*n* = 8). Different superscripts indicate significant differences between the groups (*P* < 0.05)

Repeated measures of oxygen uptake showed that time had a significant effect (*P* < 0.001), while interaction (*P* = 0.298) and group (*P* = 0.351) did not (Fig. 2a). Oxygen uptake during the initial 20 min period was elevated in the EX group compared to the CON and EXIN groups, which were nearly identical. (Fig. 2b).

**Fig. 3:**
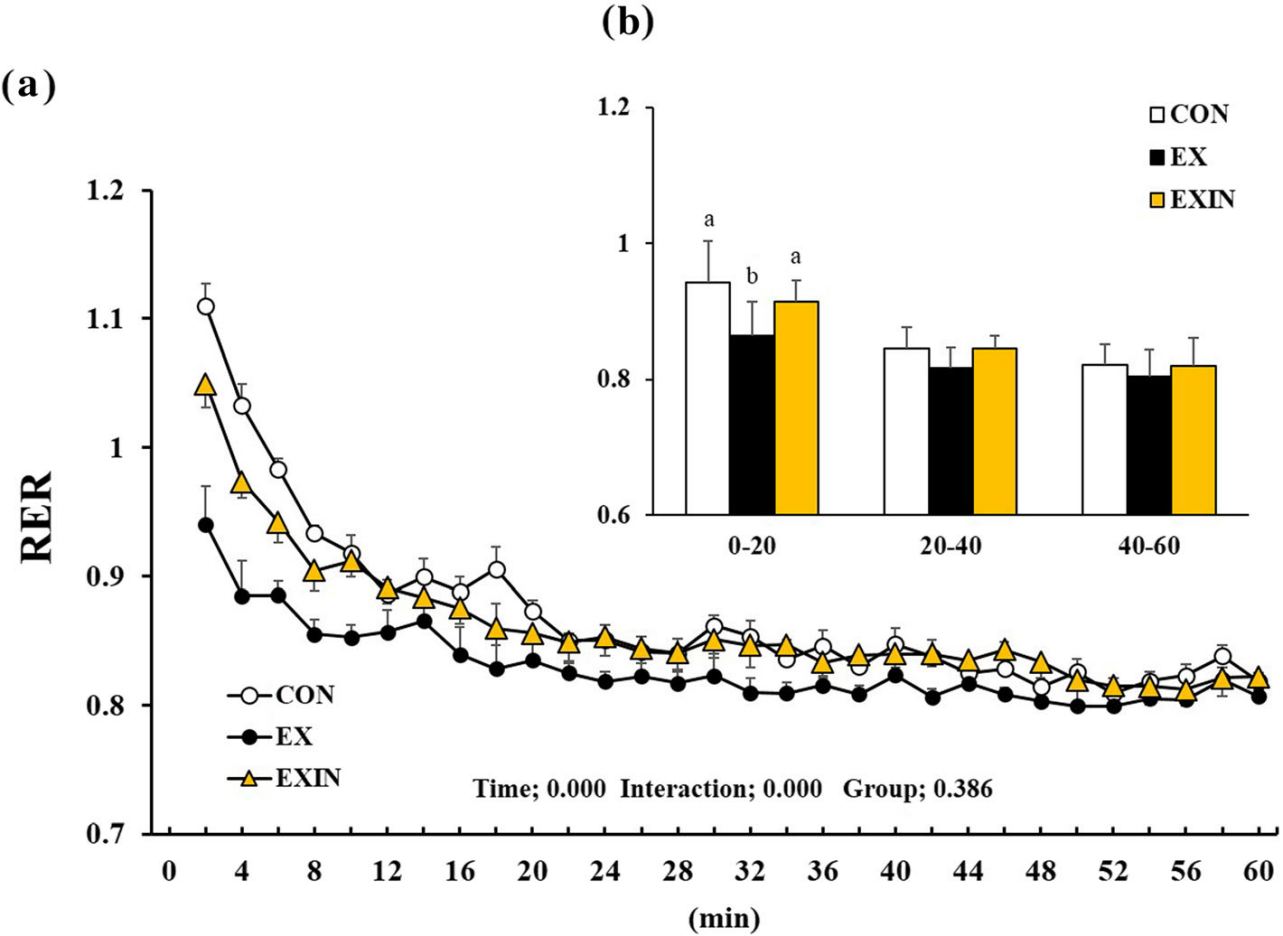
Change in respiratory exchange ratio (RER) during 1 h of exercise (**a**), the mean of the group RER for each 20 min block of exercise (**b**). Energy metabolism measured during 1 h of exercise. CON (control, *n* = 8), EX (exercise, 60% VO 2 max, *n* = 8), EXIN (Exercise + AR inhibitor, 60% VO 2 max, *n* = 8). Values are presented as means ± standard deviations (*n* = 8). Different superscripts indicate significant differences between the groups (*P* < 0.05)

Repeated measures of RER showed that time had a significant effect (*P* < 0.001). Group-by-time interaction was also significant (*P* < 0.001) but group was not (*P* = 0.386) (Fig. 3a). The RER was significantly lower in the EX group than in the CON and EXIN groups during the initial 20-min phase (Fig. 3b), while there was no difference between the CON and EXIN groups.

**Fig. 4:**
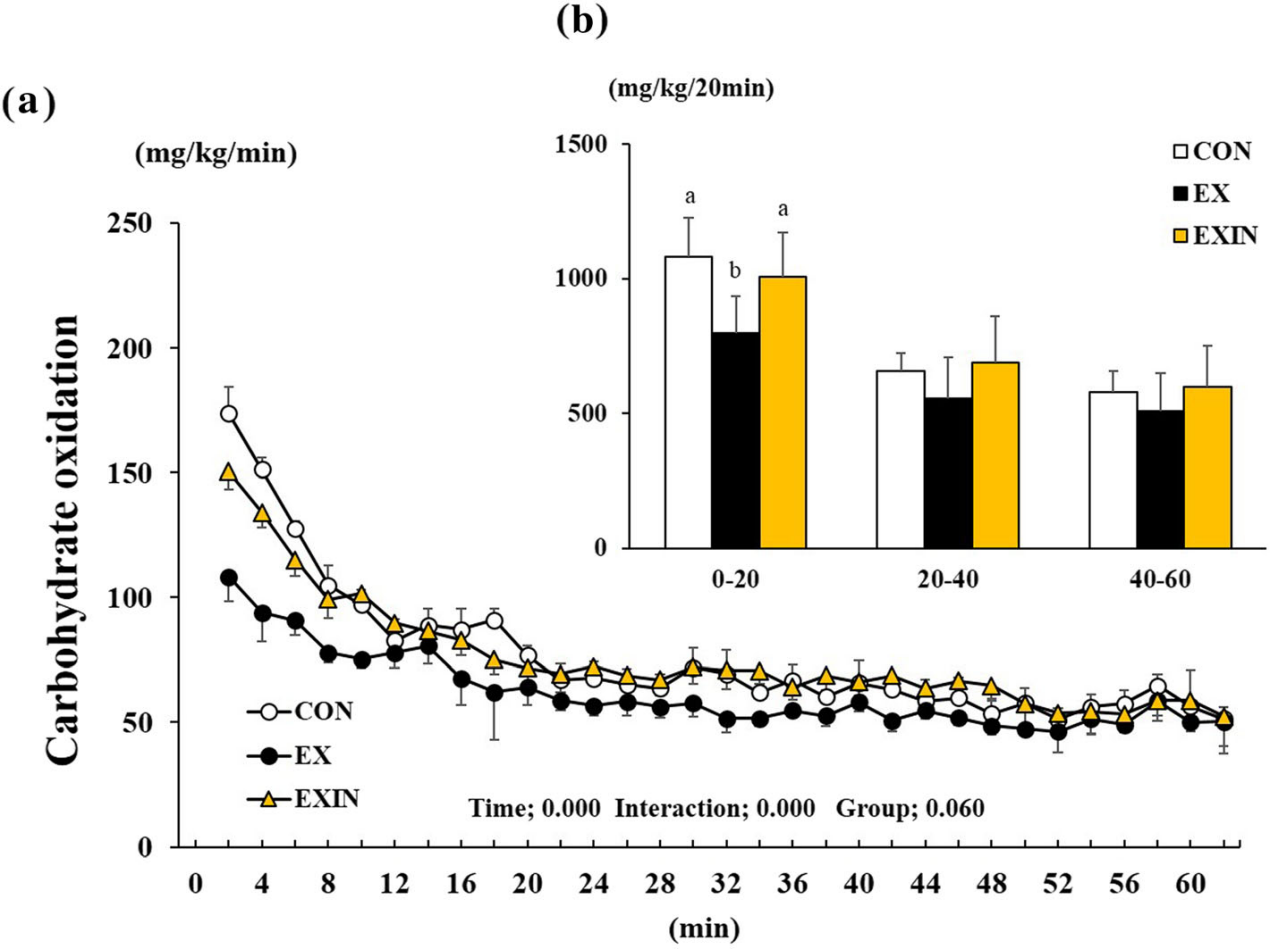
Change in carbohydrate oxidation during 1 h of exercise (**a**), the sum of each group’s carbohydrate oxidation for every 20 min block of exercise (**b**). Energy metabolism measured during 1 h of exercise. CON (control, *n* = 8), EX (exercise, 60% VO 2 max, *n* = 8), EXIN (Exercise + AR inhibitor, 60% VO 2 max, *n* = 8). Values are presented as means ± standard deviations (*n* = 8). Different superscripts indicate significant differences between the groups (*P* < 0.05)

Carbohydrate oxidation was significantly affected by time (*P* < 0.001), interaction (*P* < 0.001), and group (*P* = 0.060) (Fig. 4a). It was significantly lower in the EX group than in the CON and EXIN groups during initial 20 min phase (Fig. 4b), while there was no difference between the CON and EXIN groups.

**Fig. 5:**
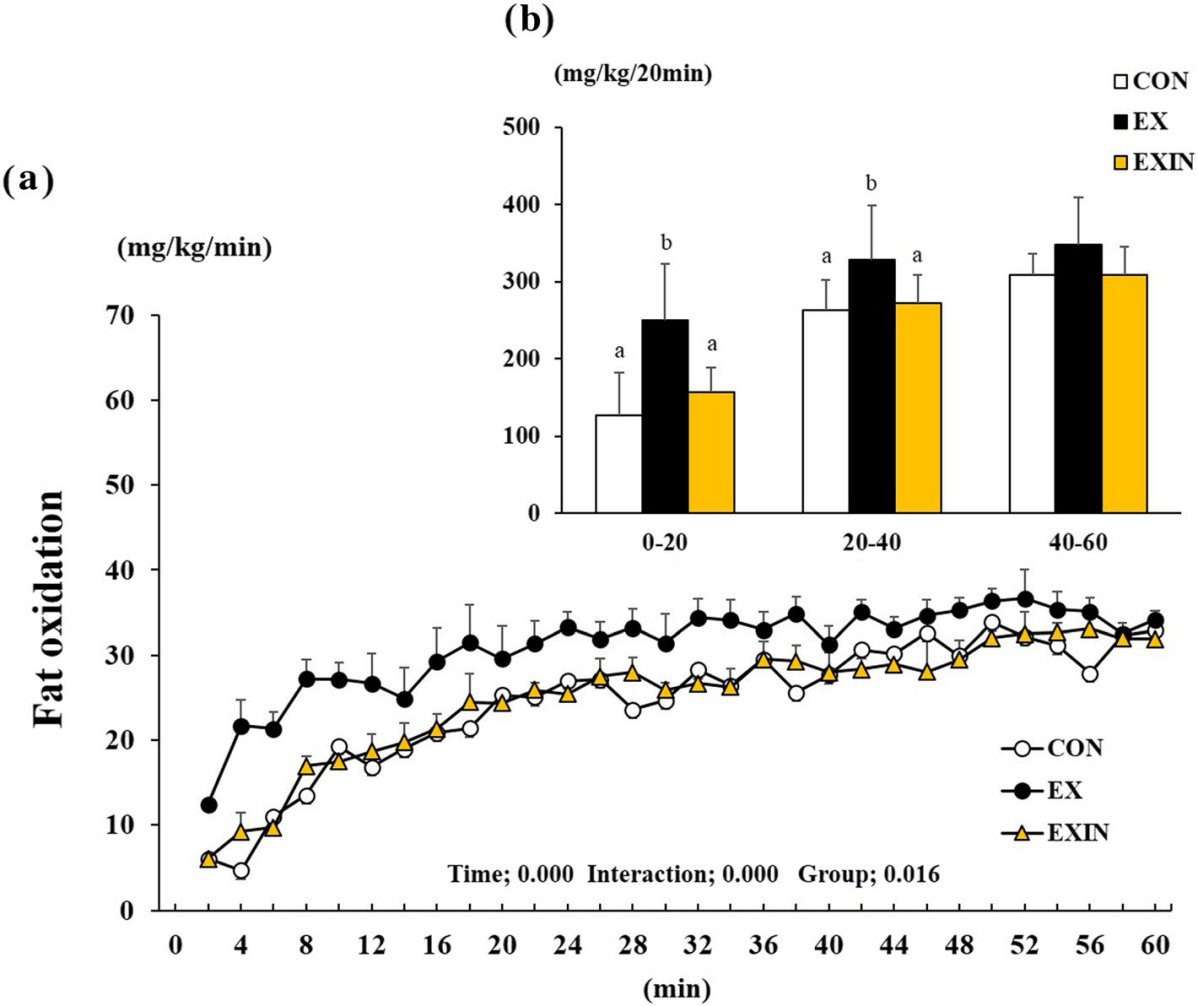
Change in fat oxidation during 1 h of exercise (**a**), the sum of each group’s fat oxidation for every 20-min block of exercise (**b**). Energy metabolism measured during 1 h of exercise. CON (control, *n* = 8), EX (exercise, 60% VO 2 max, *n* = 8), EXIN (Exercise + AR inhibitor, 60% VO 2 max, *n* = 8). Values are presented as means ± standard deviations (n = 8). Different superscripts indicate significant differences between the groups (*P* < 0.05)

Fat oxidation was affected by time (*P* < 0.001), interaction (*P* < 0.001), and group (*P* < 0.016) during the 1 h exercise (Fig. 5a). The sum of fat oxidation during the 1 h period averaged 13% higher in the EX group than in the CON and EXIN groups (data not shown). Fat oxidation increased significantly during the initial 20 min phase in the EX groups compared to that in the CON and EXIN groups (Fig. 5b) (*P* < 0.001, *P* < 0.003). In addition, the EX group showed higher fat oxidation than the CON and EXIN groups after 40 min of exercise (*P* < 0.020, *P* < 0.041). However, the EXIN group experienced the same intensity as the EX group but did not produce a high fat oxidation, like the CON group.

### Expression of FAT/CD36 and CPTІ in skeletal muscle

**Fig. 6:**
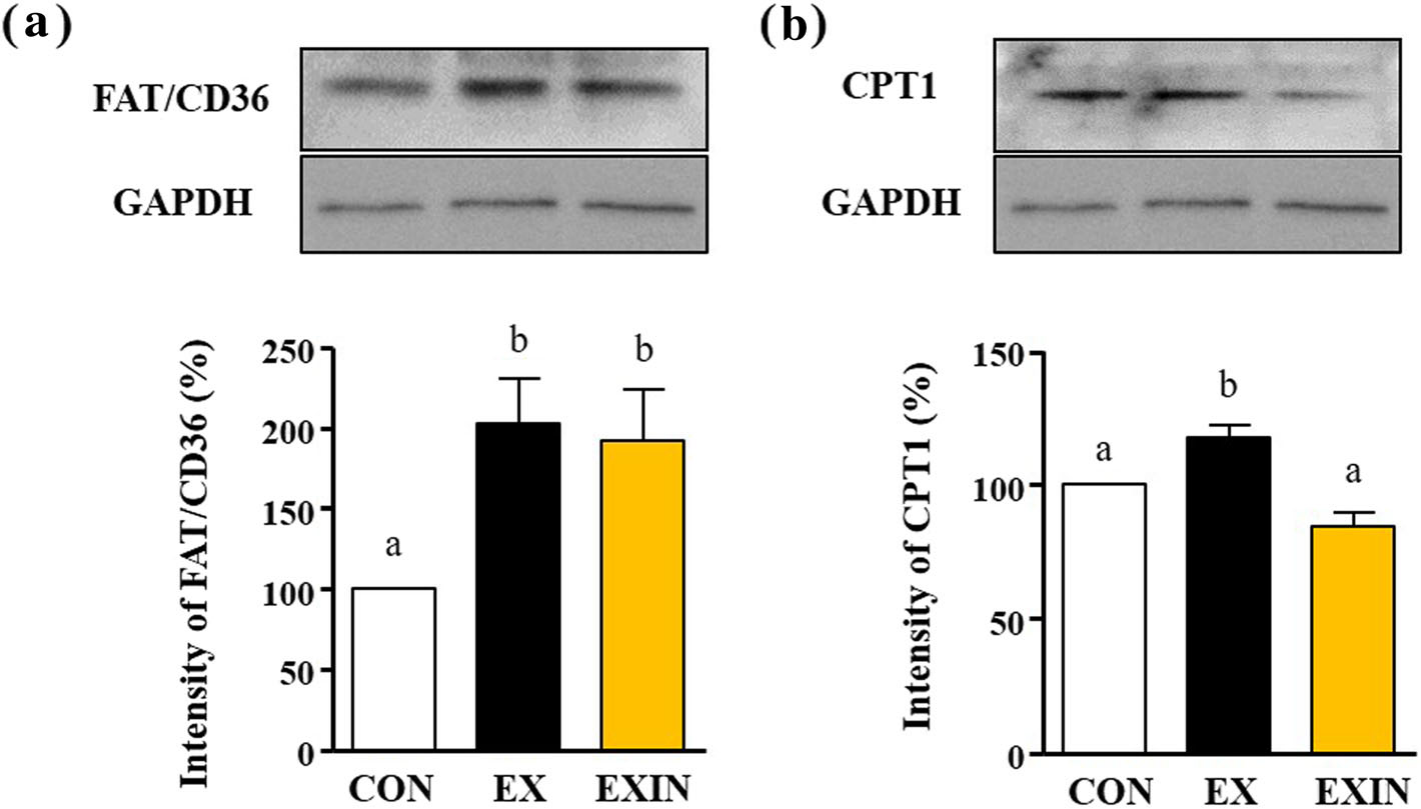
Expression levels of the (**a**) FAT / CD36 and (**b**) CPTІ in skeletal muscle analyzed by western blotting. Results are expressed as relative abundance in the EXIN group (AR inhibitor with exercise training) compared with the CON (sedentary) and EX (exercise training with placebo). Values are presented as means ± standard deviations (n = 8). Different superscripts indicate significant differences between the groups (*P* <0.05)

Western blot analysis was performed using protein obtained from the mouse skeletal muscle (soleus) samples. The FAT/CD36 protein level in the EX and EXIN groups was approximately double that of the CON group (*P* < 0.001) (Fig. 6a). The level of CPTІ increased in the EX group compared to the CON group (*P* < 0.0125) (Fig. 6b). However, CPTI in the EXIN group was significantly lower than in the EX group (*P* < 0.0069).

## Discussion

We demonstrated that a 4-week regimen of AR inhibitor treatment decreased oxygen uptake and fat oxidation relative to mice receiving placebo treatment during exercise in trained mice. On the protein expression level, we found that the AR inhibitor treatment decreased the expression of CPTІ in trained mice. Furthermore, the AR inhibitor treatment group (EXIN) showed significantly higher body weight, abdominal fat tissue weight (total fat and mesenteric fat) compared with the placebo treated exercise group. Our results suggest that the suppression of androgen hormone activity or lowering the sensitivity of AR can inhibit energy expenditure and fat oxidation by reducing CPTІ in skeletal muscle.

In this study, we observed that oxygen uptake tended to be elevated during the initial exercise phase in the EX group, higher than the CON and EXIN groups. Interestingly, the EXIN group, which exercised with the same intensity during the same period, experienced decreased oxygen uptake during exercise. The EXIN group experiencing AR blockage also showed a significant decrease in fat oxidation (12% lower than EX, data not shown) during the initial 0–20 min (*P* < 0.003) and after 20–40 min (*P* < 0.041). Furthermore, we found that body weight, abdominal fat (total and mesenteric fat) and plasma glycerol levels were significantly higher for the EXIN group compared to the EX group. Because both trained groups experienced elevated energy expenditure, the AR inhibited group may indicate lower energy efficiency and dependence on carbohydrate utilization during exercise. In additional, plasma insulin was found to be reduced in both in EX and EXIN groups due to exercise effects. However, FFA showed a significant decrease only in the EX group than in the CON group. This means that the triglycerides breakdown smoothly and FFA released into the blood is well used as energy source.

In contrast, we previously reported that application of androgen hormone (dehydroepiandrosterone, DHEA) increased energy consumption during 30 min of moderate intensity treadmill exercise [11]. In addition, DHT inhibition groups showed approximately 5.8% lower area under the curve (AUC) of fat oxidation and higher AUC of carbohydrate oxidation. This study examines the long-term use of AR blockers to better mimic hormone depletion during the aging process. As in previous studies, the inhibition of fat oxidation was similar. This study clearly confirms that chronic blockage of androgen receptors reduces energy efficiency and inhibits fat oxidation.

In this study, the FAT/CD36 and CPTІ protein levels were significantly higher in the EX group than in the CON group (*P* < 0.001, *P* < 0.0125). Continuous exercise has been reported to increase the expression of FAT / CD36 and CPTІ. These molecules transport fatty acids, mobilizing them for use as an energy source [14, 19–23]. In particular, FAT / CD36 transports fatty acids from the cell membrane to the cytoplasm and mitochondria, while CPTІ is present in the mitochondrial outer membrane and assists in translocation to the matrix [24]. The difference in CPTI expression in this study is very interesting. When ARs were inhibited, the expression of CPTI was significantly reduced (*P* < 0.0069), while FAT/CD36 expression did not decrease even if ARs is blocked. This pattern was less pronounced in the CON (non-exercise) group. In present study, AR inhibition has not affected the expression of FAT/CD36 while decreasing the expression of CPT1. This seems to be a gene that, unlike CPT1, is not affected by androgen hormones and is increased independently through exercise. Meanwhile, the decrease in CPT1 in our study seems to be due to the activation of Malonyl-CoA. Malonyl CoA is a potent inhibitor of carnitine palmitoyl transferase (CPT-1), an enzyme that controls fatty acids transport into the mitochondrion [25] (Additional file 1).

According to a recently published review of ARs, androgens bound to the ARs to stimulate the transcription of enzymes required for de novo lipogenesis and receptors that mediate the uptake of fatty acids released by lipolysis from the circulation and adipocytes [26]. Previous study that ARKO (androgen receptor knock out) mice were euphagic compared to the wild-type male controls, but also less dynamic and less oxygen consuming. Also, ARKO mice indicated that thermogenetic uncoupling protein 1 (UCP1) was lower than in wild-type group [27].

It was recently reported that androgen hormone treatment increased acyl-coenzyme A dehydrogenase long chain and hormone sensitive lipase [28]. Androgen treatment also stimulated fatty acid and triacylglycerol production, lipolysis, and cell shape reorganization [29]. In parallel, androgen hormone production increased with increasing endurance exercise capacity [30].

However, the effect of chronic AR inhibition with exercise training on LCFA transport proteins has not been elucidated, and its effect on whole body energy consumption and energy substrate composition is not yet known.

There are some limitations to our research. First, there is no group that only blocks AR. However, our study aimed at the effects of during exercise on fat metabolism and fat transport protein after blocking AR. Second, we did not measure the other gene expression related to fat metabolism. However, we confirmed that ARs blocking decreased CPT1 protein expression in the skeletal muscle and, therefore, we believe that the effect of ARs blocking on RER during exercise was due to the decrease fat utilization. In addition, many studies have reported that FAT / CD36 and CPT1 play a pivotal role in fatty acids transport and are highly correlated with whole body fat oxidation. Third, we know that all chemical inhibitors are not specific, so we think that can not rule out the metabolic changes caused by other effects of inhibitors. It is also believed that additional studies will be needed to clarify the effectiveness of the inhibitor. In future investigations, it would be necessary to elucidate the effects of AR inhibition on the resting metabolism and a clear mechanism of fatty acids transport proteins.

## Conclusions

We observed that chronic treatment of mice with AR inhibitor while exercise training reduced whole-body fat utilization and energy efficiency in male mice. Furthermore, AR blockade inhibited CPTІ production in skeletal muscle. Our results suggest that a can decrease in androgen concentration or androgen receptor sensitivity affects exercise capacity by downregulating CPTІ. Reduction of CPTI results in inhibition of fat oxidation and reduced energy efficiency by depriving skeletal muscle mitochondria of LCFA energy sources.

## Supplementary information

**Additional file 1: Figure S1.** Expression levels of the FATP and FABP in skeletal muscle analyzed by western blotting. Results are expressed as relative abundance in the EXIN group (AR inhibitor with exercise training) compared with the CON (sedentary) and EX (exercise training with placebo). Values are presented as means ± standard deviations (*n* = 8).

## Data Availability

The data used to support the findings of this study are included within the article or available from the corresponding author upon request.

## Abbreviations

AR: Androgen receptors
AUC: Area under the curve
BAT: Brown adipose tissues
CPTI: Carnitine palmitoyltransferase I
DHEA: Dehydroepiandrosterone
FAT/CD36: Fatty acid translocase/cluster of differentiation 36
FFA: free fatty acids
LCFA: Long-chain fatty acid
RER: respiratory exchange ratio
