# Supplementary figures and images for: Inhibition of androgen receptor can decrease fat metabolism by decreasing carnitine palmitoyltransferase I levels in skeletal muscles of trained mice
Source: Nutr Metab (Lond). 2019 Nov 27;16:82. doi: 10.1186/s12986-019-0406-z (PMC6880567; doi:10.1186/s12986-019-0406-z)

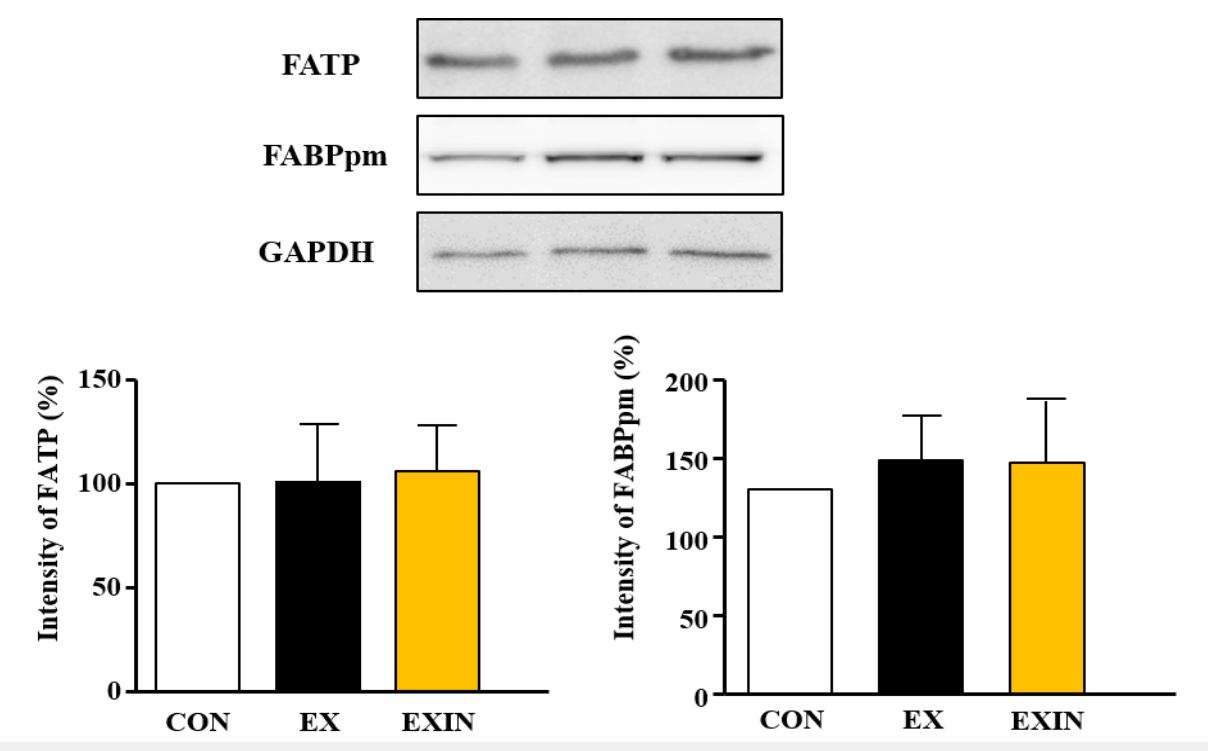

Supplement: Supplementary file 1 — Additional file 1: Figure S1. Expression levels of the FATP and FABP in skeletal muscle analyzed by western blotting. Results are expressed as relative abundance in the EXIN group (AR inhibitor with exercise training) compared with the CON (sedentary) and EX (exercise training with placebo). Values are presented as means ± standard deviations (n = 8). [file 12986_2019_406_MOESM1_ESM.jpg]
